# Pessimism and risk of death from coronary heart disease among middle-aged and older Finns: an eleven-year follow-up study

**DOI:** 10.1186/s12889-016-3764-8

**Published:** 2016-11-17

**Authors:** Mikko Pänkäläinen, Tuomas Kerola, Olli Kampman, Markku Kauppi, Jukka Hintikka

**Affiliations:** 1 Department of Psychiatry, Päijät-Häme Central Hospital, Keskussairaalankatu 7, FI-15850 Lahti, Finland; 2 Department of Internal Medicine, Päijät-Häme Central Hospital, Lahti, Finland; 3 Department of Psychiatry, Seinäjoki Hospital District, Seinäjoki, Finland; 4 University of Tampere, School of Medicine, Tampere, Finland

**Keywords:** Pessimism, Optimism, Cardiovascular disease risk, Coronary heart disease, Mortality, Life Orientation Test, Revised

## Abstract

**Background:**

Mortality from coronary heart disease (CHD) remains at quite notable levels. Research on the risk factors and the treatment of CHD has focused on physiological factors, but there is an increasing amount of evidence connecting mental health and personality traits to CHD, too. The data concerning the connection of CHD and dispositional optimism and pessimism as personality traits is relatively scarce. The aim of this study was to investigate the connection between optimism, pessimism, and CHD mortality.

**Methods:**

This was an 11-year prospective cohort study on a regional sample of three cohorts, aged 52–56, 62–66, and 72–76 years at baseline (*N* = 2815). The levels of dispositional optimism and pessimism of the study subjects were determined at baseline using a revised version of the Life Orientation Test (LOT-R). Eleven years later, those results and follow-up data about CHD as a cause of death were used to calculate odds. Adjustments were made for cardiovascular disease risk.

**Results:**

Those who died because of CHD were significantly more pessimistic at baseline than the others. This finding applies to both men and women. Among the study subjects in the highest quartile of pessimism, the adjusted risk of death caused by CHD was approximately 2.2-fold (OR 2.17, 95 % CI 1.21–3.89) compared to the subjects in the lowest quartile. Optimism did not seem to have any connection with the risk of CHD-induced mortality.

**Conclusions:**

Pessimism seems to be a substantial risk factor for death from CHD. As an easily measured variable, it might be a very useful tool together with the other known risk factors to determine the risk of CHD-induced mortality.

## Background

Coronary heart disease (CHD) is still the leading cause of mortality, despite growing knowledge of its risk factors and the new treatments available [1]. According to the latest statistics, CHD causes about 200 deaths per 100,000 annually in industrialized countries (e.g. in 2013 193.3/100,000 in the United States and 193.6/100,000 in Finland) [2, 3].

The majority of those with CHD have at least one of the four most important physiological risk factors (diabetes, hypertension, smoking, or elevated lipids) [4]. Some CHD patients seem to have no recognizable physiological risk factors and there are also many people with one or more physiological risk factors and still no CHD, which supports the influence of psychosocial factors in the pathogenesis of CHD.

The heart has always been described as a centre of psychosocial health and emotions in the history of art and culture. The scientific connection between psychosocial health and the heart was studied for the first time in 1937, when Benjamin Malzberg investigated the connection between involutional depression and the elevated rate of cardiovascular deaths [5]. Since then, there has been only limited interest in this subject. Recently, however, the scientifically significant linkage between psychosocial health and the heart has been proved. For example, in the INTERHEART study, psychosocial factors such as depression and psychosocial stress were found to be one of the most significant risk factors for myocardial infarction [6, 7], and the American Heart Association has stated that depression is an independent risk factor for a poor prognosis following an acute coronary syndrome [8].

The connection of cardiovascular health with optimism and pessimism is under increasing investigation. Links have been found between optimism/pessimism and, for example, the risk of strokes [9], the status of the major arteries [10], the risk of incident heart failure [11], the recovery of patients from coronary artery bypass surgery [12–14], and the incidence of CHD [15–18]. All of these studies have stated that optimism (or the lack of pessimism) is connected with better cardiovascular outcomes. Even an optimistic attitude towards one’s cardiac health, whether justifiable or not, seems to be an independent factor that enhances the health of the cardiovascular system [19]. Recently, a large review concerning positive psychological constructs and health outcomes in patients with cardiovascular disease was published [20], and some years earlier another review was made about optimism and physical health with a notable section on the health of the heart [21]. Nevertheless, none of these studies or parts of the reviews pays any attention to the mortality caused by CHD.

The results of most of the studies concerning the connection of the optimism construct and cardiovascular health suggest that optimism or a low level of pessimism protects from cardiac problems. When searching for literature concerning the linkage between optimism, pessimism, and the risk of cardiovascular death, we could only find four studies; they had contradictory results and none of them were included in the reviews mentioned earlier. According to one prospective study, one-dimensionally assessed optimism seemed to diminish all-cause mortality, mostly by preventing cardiovascular deaths [22]. In another study, one-dimensionally assessed dispositional optimism protected men from cardiovascular death [23]. One study with only female study subjects found that optimism diminished mortality related to CHD [16]. However, in a cross-sectional study where optimism was also assessed as a single factor – with optimism and pessimism as opposites – optimism seemed to increase both cardiovascular mortality and all-cause mortality [24].

We did not find any prospective studies on general population samples where the risk of death caused by CHD was evaluated separately for optimism and pessimism. In addition, in earlier studies single-factor optimism (i.e. the one-dimensionally assessed bipolar factor with optimism and pessimism as opposites) seemed to have a controversial connection to CHD-related deaths. Therefore, we conducted this 11-year follow-up study on middle-aged and older Finnish men and women in which we assessed whether optimism and pessimism as independent variables are true protective or risk factors for CHD mortality.

## Methods

The GOAL (Good Ageing in Lahti region) study was started in the district of Lahti, Finland in 2002. Its aim was to find out ways to improve the health and well-being of the local aging population in the future. Stratified (age, sex, municipality) random samples of men and women born in 1926–30, 1936–40, and 1946–50 were drawn from the population register of all 14 municipalities in the Lahti region. A total of 4272 subjects were invited and 2815 (66 %) participated. At baseline, the study subjects filled in questionnaires concerning their current status of life (e.g. socioeconomic status, psychosocial background, health, and lifestyle). Levels of blood glucose and blood total and high-density lipoprotein (HDL) cholesterol were determined with standardized methods. The blood pressure of study subjects was measured at baseline three times and the average was documented. The smoking habits were also documented, and the patients were asked about their use of drugs for hypertension and/or diabetes. Finally, the study subjects were asked at baseline whether they had CHD diagnosed by a doctor.

The study subjects filled out the revised version of the Life Orientation Test (LOT-R) to measure their optimism and pessimism. The original Life Orientation Test (LOT) was developed in the mid-1980s in order to investigate the effects of dispositional optimism on the selfregulation of behavior in a wide variety of domains, some of them health-related [25]. In 1994, the test was re-evaluated and revised (LOT-R) by Scheier, Carver, and Bridges [26] to focus its item content more closely on expectancies of the future. The questionnaire includes four fillers (which were disregarded when determining the level of optimism and pessimism) and six actual statements, of which three are worded positively to indicate optimism (e.g., “In uncertain times, I usually expect the best”) and three are worded negatively for pessimism (e.g., “If something can go wrong for me, it will”). The respondents are asked to indicate how well the statements describe them in general, as expressed on a scale from 0 (not at all) to 4 (very much so). A higher score refers to greater optimism or greater pessimism depending on the statement.

Even if both LOT and LOT-R were thought to be one-dimensional scales, later studies have suggested that they may have two separate independent dimensions: optimism and pessimism. Separating optimism and pessimism, at least when they are measured by using LOT or LOT-R, has led to the better prediction of outcomes in many studies [27–31]. In our previous work, we also found that in this age-specified general population sample, LOT-R is a scale with two independent subscales [32], and the use of the bipolar model (i.e. optimism and pessimism as one variable) would have hidden some of results found in our study. Thus, in the analyses of this study, we used independent subscale scores separately for the optimism and pessimism components. They were named optimism and pessimism, respectively.

Eleven years after the baseline of the study, on 31 December 2013, we could find 2719 (97 %) of the original 2815 study subjects from official statistics of the study area. Fifty subjects had to be excluded due to severe deficiencies in baseline data, diminishing the study group to 2669 subjects. Of these, 523 had died between the baseline and 31 December 2013. Those whose underlying cause of death was other than CHD were excluded (*n* = 402). Therefore, the final study sample included 2267 study subjects, of whom 121 had died from CHD during the 11-year follow-up, meaning that 2146 were still alive.

In this study, we calculated a general cardiovascular disease risk score (CVD risk score) for each participant. This scoring has been developed as a part of the Framingham Heart Study for use in primary care [33]. It is a sum of sex-specific scorings of the following general risk factors for cardiovascular diseases: age, total cholesterol, HDL cholesterol, systolic blood pressure, smoking, and diabetes. The scoring of systolic blood pressure in the CVD risk algorithm depends on whether the subject is treated for hypertension or not. Smoking status was recorded as regular smoking or not, and this information was ascertained by self-report. Diabetes was defined as fasting glucose ≥7 mmol/L, the use of insulin, the use of oral antidiabetic drugs, or a self-report of having diabetes diagnosed by a doctor.

In statistical analyses, we used the chi-squared test for categorical variables. For comparing continuous variables, we used the nonparametric Mann–Whitney *U* test and Kruskal-Wallis tests. Finally, we calculated logistic regression models to determine adjusted odd ratios for the risk of death from CHD. Adjustments for age and sex were not made because the CVD risk scorings we calculated were already sex-specific and they also included age as one of the risk factors.

## Results

Men died from CHD more often than women during the follow-up (87/1047 (8.3 %) vs 34/1220 (2.8 %), chi-squared 34.01, *p* < 0.001). Furthermore, those who died from CHD were older at baseline (mean 70.0 years (SD 6.2) vs 62.5 years (SD 7.8), Mann–Whitney *U* test *p* < 0.001).

There were no differences between men and women in optimism (LOT-R subscale score mean (SD): 8.34 (2.10) vs 8.40 (2.08), Mann–Whitney *U* test *p* = 0.70) or in pessimism (3.85 (2.67) vs 3.80 (2.61), *p* = 0.83, respectively). No differences were found in optimism between age groups (aged 52–56 vs 62–66 vs 72–76 years: 8.26 (2.17) vs 8.38 (2.05) vs 8.53 (2.05), Kruskal-Wallis test *p* = 0.10), but those of higher age were more pessimistic (3.34 (2.68) vs 3.86 (2.57) vs 4.42 (2.56), *p* < 0.001, respectively).

Those who died from CHD during the 11-year follow-up had been significantly more pessimistic at baseline than the subjects who were still alive (LOT-R subscale score mean (SD): 4.78 (2.41) vs 3.77 (2.64), Mann–Whitney *U* test *p* < 0.001), while in optimism, there was no difference (LOT-R subscale score mean (SD): 8.40 (2.17) vs 8.37 (2.09), *p* = 0.98, respectively). These findings apply to both genders (Table 1).

**Table 1:** Risk factors at baseline and death from coronary heart disease during the 11-year follow-up in men and women

	Men					Women				
	Death from coronary heart disease				Mann–Whitney	Death from coronary heart disease				Mann–Whitney
	Yes		No		*U* test	Yes		No		*U* test
	*N* = 87		*N* = 960			*N* = 34		*N* = 1186		
	Mean	SD	Mean	SD	*p*-value	Mean	SD	Mean	SD	*p*-value
Total cholesterol (mmol/L)	5.26	1.08	5.67	1.09	0.01	5.52	1.44	5.90	1.03	0.03
HDL cholesterol (mmol/L)	1.21	0.30	1.37	0.36	<0.001	1.47	0.41	1.65	0.45	0.04
Systolic blood pressure (mmHg)	148	23	146	18	0.39	152	25	145	20	0.09
Blood glucose (mmol/L)	6.48	1.86	5.86	1.27	0.001	5.91	1.00	5.51	1.16	0.001
CVD risk score	18.2	3.5	15.9	3.7	<0.001	18.0	3.5	14.1	4.2	<0.001
LOT-R optimism score	8.37	2.15	8.34	2.11	0.97	8.45	2.31	8.40	2.07	0.87
LOT-R pessimism score	4.56	2.51	3.78	2.68	0.008	5.34	2.03	3.75	2.61	<0.001
	%		%		Chi-squared test *p*-value	%		%		Chi-squared test *p*-value
CHD at baseline	42.5		9.4		<0.001	51.4		5.7		<0.001
Use of drugs for hypertension	42.5		27.0		0.002	28.6		60.0		<0.001
Use of drugs for diabetes	19.5		5.2		<0.001	14.3		3.4		0.001
Regular smoker	20.7		15.9		0.25	2.9		9.5		0.18

*HDL* high-density lipoprotein, *CVD* cardiovascular disease, *LOT-R* Life Orientation Test – Revised, *CHD* coronary heart disease

Those men and women who had died from CHD during the follow-up had had lower baseline total and HDL cholesterol levels and higher blood glucose levels than those men and women who were still alive, and their total general CVD risk scores were higher. Not surprisingly, those men and women who died from CHD during the follow-up had at baseline more often reported having CHD diagnosed by a doctor. They also used medication for hypertension and diabetes more often than the other men and women (Table 1).

Finally, we calculated a logistic regression model for the risk of death from CHD. Instead of using separate single risk factors, we included only the baseline pessimism subscale score, the presence of CHD, and the general CVD risk score (which includes the most significant physiological risk factors for CHD in itself) in the model. Pessimism was associated independently statistically significantly with the risk of death from CHD (Table 2; Model 1). To highlight the significance of pessimism as a risk factor for CHD-induced death, we compared the highest and the lowest quartiles of pessimism in a similar model. Those who were in the highest quartile of pessimism had nearly a 2.2-fold higher adjusted odds ratio for death from CHD during the 11-year follow-up period when compared to those in the lowest quartile of pessimism (Table 2; Model 2).

**Table 2:** Adjusted risk of death from coronary heart disease during the 11-year follow-up

	Risk of death from coronary heart disease					
	Model 1			Model 2		
	OR	95 % CI	*p*-value	OR	95 % CI	*p*-value
CHD at baseline	8.09	5.35–12.23	<0.001	7.41	4.38–2.53	<0.001
CVD risk score at baseline	1.26	1.19–1.33	<0.001	1.30	1.21–1.39	<0.001
Pessimism (score)	1.08	1.00 –1.16	0.039	…	…	…
Pessimism (quartiles; highest/ lowest)	…	…	…	2.18	1.21–3.89	0.010

Model 1 = the pessimism score is included as a continuous variable

Model 2 = the pessimism score has been divided into quartiles and the highest quartile has been compared with the lowest

Both models include the presence of CHD and the CVD risk score at baseline

*OR* adjusted odds ratio, *95 % CI* 95 % confidence interval, *CHD* coronary heart disease, *CVD* cardiovascular disease

## Discussion

Our main finding was that pessimism is a strong independent risk factor for death from CHD. The magnitude of the result seems to be quite similar when compared to the three earlier studies we found on this subject [16, 22, 23]. Nevertheless, results from those other studies cannot be directly compared to our findings because in those studies the optimism construct was determined as a bipolar single factor, whereas we used separate optimism and pessimism variables. In our study, optimism did not associate with the mortality rates induced by CHD.

Those with higher scores on the pessimism subscale at baseline may have had more physiological risk factors of CHD already at the beginning of the 11-year follow-up and one might think that awareness of those risk factors could be one reason for pessimism. However, it has been demonstrated that personality traits evolve at a relatively early age and after that they are very stable. For example, bad news about one’s health seems to have no effect on the LOT-R scores [34, 35].

Our result – pessimism being the only variable out of optimism and pessimism that mediates the effect of the optimism construct on the risk of CHD-induced death, while the optimism has no influence at all – is not unique. For example, in the review by Rasmussen et al. it was also speculated that the presence or absence of pessimism alone might determine the effect of the optimism construct on cardiac health, regardless of optimism [21]. This emphasizes the stance that the optimism construct should be seen to include two separate and independent dimensions, optimism and pessimism, instead of one continuum with two poles. This means that people should not be categorized as “optimists” or “pessimists”. This theory is supported by several other studies, too [27–31].

Optimism in the scientific sense focuses on expectancies of the future, which links it to expectancy-value models of motivation [36]. In other words, if a person is optimistic about something she/he wants to achieve, she/he may consider that goal achievable, which in turn may help and motivate behaviour in a way that enables reaching that goal. In pessimism, the connection is logically converse: if a person is pessimistic about something she/he wants to achieve, she/he may consider that goal as somehow impossible or at least improbable, which may diminish the efforts made to accomplish the goal. For example, when studying the optimism construct and cardiac health, a high level of optimism has been found to associate with a healthier lifestyle, for example with higher vegetable, fruit, and whole-grain bread consumption; higher physical activity; lower smoking rates; a healthier diet; a healthy lipid profile; and a lower body mass index, which all decrease the incidence of CHD [37–39]. These examples are connected with behaviour, which in turn is thought to be affected by the optimism construct. On the other hand, a high level of pessimism has been linked with several factors that have effects on cardiac health, i.e. elevated inflammation markers, endothelial dysfunction, and shorter telomere length [40, 41]. These factors cannot as clearly be seen as direct consequences of behaviour.

The optimism construct seems to have a clear impact on physiological health and CHD mortality even after adjustments for the well-known classical risk factors of cardiovascular diseases. This finding suggests that our knowledge about the connection between the optimism construct and physical health is far from complete. Separating optimism and pessimism seems to highlight that pessimism may be the variable in the optimism construct that mediates the effect, and this separation may be of benefit in studying this topic in the future.

One strength of this study is its design. The study group was selected randomly and it was constituted of equal numbers of both sexes and representatives of all the age groups invited, so the study group can be seen as comprehensive. Eleven years is a relatively long time and it seems to be enough for the detectable and statistically significant differences in CHD-induced mortality to appear. The fact that the study was prospective makes it more reliable. In our study, life orientation was measured using the well-known test pattern of the LOT-R, and optimism and the pessimism were seen as different variables, which seems to clarify the results.

There are a few limitations in this study as well. It is probable that poorly functioning and institutionalized persons had a lower participation rate than community-dwelling subjects. It is also probable that the incidence of CHD-induced death would have been higher in those populations. At the same time, it is not known whether there are any differences in pessimism between these groups and rest of the population. Much of the data used in this study is based on the questionnaires filled out by the study subjects themselves, so there might be some inconsistency between the answers and the reality in the questions concerning, for example, smoking habits and use of the medications.

## Conclusions

Pessimism seems to be quite a significant risk factor for death from coronary heart disease both in men and women, while optimism does not protect from it. Assessing optimism and pessimism as separate entities improves the prognostic values of the connection between these personality traits and coronary heart disease. The level of pessimism can be measured easily and non-invasively and it might be a very useful tool together with the other known risk factors to determine the risk of CHD-induced mortality.

## Abbreviations

CHD: Coronary heart disease
CVD risk score: General cardiovascular disease risk score
GOAL: Good Ageing in Lahti region study
HDL: High density lipoprotein
LOT: Life orientation test
LOT-R: Revised version of the Life Orientation Test
